# Understanding energy system dynamics in a changing climate: Insights for first responders and risk managers

**DOI:** 10.1016/j.isci.2025.113320

**Published:** 2025-08-08

**Authors:** Jonathan Mille, Danielle Charlton, Marleen C. de Ruiter, Raphael Stevens, Muki Haklay, Stephen J. Edwards

**Affiliations:** 1 Department of Earth Sciences, University College London (UCL), London, UK; 2 UCL Climate Action Unit, University College London (UCL), London, UK; 3 Auckland CBD, GNS Science Te Pῡ Ao 12 Madden St, Auckland 1010, New Zealand; 4 Institute for Environmental Studies (IVM), Vrije Universiteit Amsterdam, Amsterdam, the Netherlands; 5 Momentum Institute, Paris, France; 6 Department of Geography, University College London (UCL), London, UK

**Keywords:** Earth sciences, Climatology, Energy resources, Energy policy, Energy Resources, Energy systems, Energy management

## Abstract

Climate change and the evolving global energy landscape pose intertwined escalating challenges for risk management, driven by diminishing fossil fuel reserves, mineral dependencies, climate variability, and geopolitical shifts. This article explores the systemic risks and cascading vulnerabilities embedded within energy system changes in a climate change context, focusing on their implications for first responders and disaster risk reduction strategies. The perspective highlights dependencies on fossil fuels, critical minerals, and global supply chains, alongside the new range of risks introduced by renewable energy transition and climate-induced disruptions. Structural and necessary changes required for electrification and decarbonization may complicate energy stability and infrastructure resilience. Particular attention is paid to the operational blind spots that may affect first responders and emergency managers. The article advocates for the inclusion of energy system dynamics in multi-risk assessments and emphasizes the importance of cross-sector collaboration to enhance systemic resilience. By synthesizing interdisciplinary evidence, it provides a strategic foundation for first responders, risk managers, and policymakers to anticipate and adapt to the interlinked risks of a shifting energy paradigm.

## Graphical abstract

**Figure:**
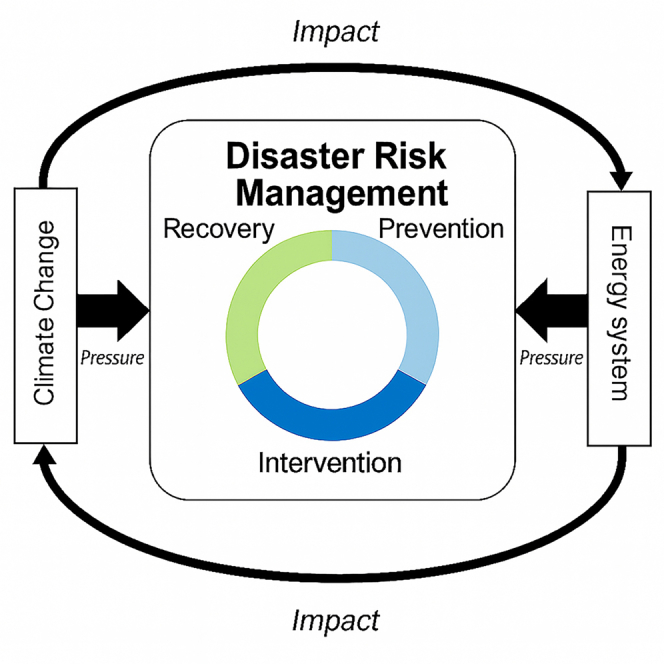

## Introduction

The conflict in Ukraine has intensified concerns about reduced gas and oil supplies from Russia and has prompted political leaders to reconsider issues of sovereignty and energy security. The consequences of this event, still unfolding at the time of writing, are redrawing the global map of energy distribution and supply.^1^ However, whether triggered by conflict or extreme natural events such as earthquakes or tsunamis, disruptions to energy supplies can have multiple and temporal and spatial repercussions.^2^ Although such events have significant immediate impacts, they typically represent episodic disruptions in the broader historical context of energy supply. In contrast, deeper mechanisms operate over longer time scales and can have lasting impacts on the global energy systems.^3^^,^^4^ These mechanisms can induce systemic and enduring impacts to human societies. While energy supply and demand are often managed at the national level, global energy supply chains increasingly influence local costs, infrastructure stability, and risk management strategies.^5^

National risk systems are now deeply interconnected with global energy trends, rendering them vulnerable to fluctuations in global energy availability.^6^ Recent analyses in gray literature^7^^,^^8^ highlight the growing need for risk managers to incorporate energy-specific insights into broader systemic risk assessments, reflecting on the interconnected risks between energy systems, supply chains, and resilience strategies. This article will therefore focus on the dynamics and variability of energy supply, investigating the limits as well as potential long-term disruptions of energy supplies as a systemic threat, one that may be underappreciated and warrants greater attention from risk managers and policymakers. In this article, we consider risk managers to include first responders, frontline professionals, and operational personnel responsible for designing and implementing prevention, intervention, and recovery strategies during extreme events.

Human systems are fundamentally dependent on continuous energy flows to maintain their structure and function.^9^^,^^10^^,^^11^^,^^12^ In some parts of the world, a constant and growing supply of energy is part of everyday life and can be taken for granted. Yet, recent crises from climate change to COVID-19 and the Ukraine conflict have exposed critical energy dependencies and vulnerabilities, especially within global energy supply chains.^13^^,^^14^ Understanding these phenomena is critical to assessing cascading risks within energy systems, as they can trigger widespread and interconnected failures across supply chains and infrastructure, ultimately challenging resilience and adaptive capacity.^15^^,^^16^ The current energy system, with its extensive infrastructures and globalized supply chains, stands at a critical turning point and may face an unprecedented supply crisis.^17^^,^^18^^,^^19^ Meanwhile, efforts to accelerate the transition toward electrification and greater energy sobriety are intended to reduce greenhouse gas emissions and address climate change.^20^

Systems heavily reliant on fossil energy are inherently vulnerable: any disruption in its energy supply can trigger wide-ranging and cascading effects.^4^^,^^21^^,^^22^ Since risk management strategies themselves depend on reliable energy systems for prevention, response, and recovery, changes in energy availability will also, and inevitably, affect how risk is managed. Despite the media attention surrounding energy, public and non-experts professional understanding of how energy systems function, how they are sourced, transported, and regulated, remains limited. This includes among first responders and risk managers. To date, little research has been found to assess the understanding of energy supplies and systems by risk managers or key decision-makers. A potential knowledge gap may exist, and it is crucial to develop greater knowledge and discussion on the issues of energy prospects and their impact on future risk management strategies in a changing environmental and energy context.

In recent years, there has been growing attention to move risk assessments from a single risk to a multi-risk approach.^23^^,^^24^^,^^25^ Although challenges remain in operationalizing this shift,^23^^,^^26^^,^^27^^,^^28^ the ongoing transformation of the energy landscape presents a timely opportunity to adopt more systemic risk management strategies. These strategies must focus on critical resources that underpin every phase of disaster management, from prevention to recovery.

As systemic changes in both the type and volume of energy supply reshape human systems, risk managers will need to take a more integrated approach. They must understand the dynamics of energy systems and proactively develop mitigation strategies to manage energy supply variability. Integrating energy into risk management introduces a new dimension of systemic vulnerability and fosters the development of innovative multi-risk scenarios, especially in the context of climate change, where physical hazards are themselves evolving. Given that access to energy is essential to the functioning, security, and stability of modern societies, all potential sources of energy disruption must be systematically addressed, even the variations occurring at a global scale.

The aim of this article is to offer risk managers and first responders a non-exhaustive yet strategic overview of the limitations of global energy systems in the 21st century, and the implications for systemic resilience. This article provides a novel contribution to the field of risk management by foregrounding structural changes in global energy systems, including fossil fuel depletion, mineral dependencies, and energy transition uncertainties, as central components of future risk landscapes. While climate change is widely acknowledged as a risk driver, this study argues that the intertwidness between climate change and energy variability creates another type of risk in its own right and as such must become a core pillar of risk assessment and strategy. To our knowledge, this is one of the first studies to explicitly connect systemic energy supply dynamics with operational and strategic challenges for first responders and risk managers.

Methodologically, the article is grounded in a narrative literature review. It synthesizes peer-reviewed research, gray literature (e.g., International Energy Agency reports), and conceptual models to build a new framework for energy-informed risk management. This framework posits that future Disaster Risk Reduction (DRR) and emergency preparedness strategies must be reframed within the structural boundaries of an evolving energy paradigm and a climate change context. The article outlines plausible energy transition pathways, from rapid low-carbon deployment to delayed or resource-constrained shifts, and explores their implications for resilience planning. These scenarios offer entry points to guide risk managers in adapting tools and strategies for increasingly complex energy futures.

## Systemic challenges

Within the last 200 years, human systems have complexified.^29^ The use of energy has expanded the spatial scales of these systems and their supply chains, which are now globalized at all levels: economic, financial, industrial, and even social. This has brought many benefits as well as undesired side effects.^30^ One significant consequence is the emergence of a new spatial scale of risks: global systemic risks.^25^^,^^31^^,^^32^^,^^33^^,^^34^^,^^35^ Due to the globalization and interconnectedness of human systems, incidents in one region can rapidly impact others, as evidenced by the 2008 financial crisis or the COVID-19 pandemic.^36^^,^^37^^,^^38^ Despite their significant impacts, global systemic risks are challenging to perceive, grasp and evaluate on an individual scale.^39^ Beyond the question of geographical scale, global systemic risks also present a challenge in terms of governance.^31^^,^^32^. As some aspects of human systems have reached a global scale, actors and stakeholders belong to different sub-systems, such as energy systems, that are now spread over several continents. These subsystems, including energy systems, may have different types of governance, societies, laws, and regulations, which makes systemic risk difficult to assess and manage. The implementation of management strategies for global systemic risks is therefore also complex due to the interdependence and multidisciplinarity of the elements that constitute these strategies. The interconnectedness of global systems also confers a complexity to systemic risks, where multiple tipping points can exist at different temporal and spatial scales. Among these global and interconnected tipping points, variations in energy supply are among the potential systemic risks.^40^

The world energy system has a very special role. It is both the basis for the maintenance and development of current human systems but also a factor of dependence and therefore a source of vulnerability. Thus, the evolution of the global energy system has repercussions on human systems and can be a factor of risk if it deteriorates. Moreover, among existing services within human systems, risk management strategies, established to protect these very systems, depend on the energy supply of the systems in which these strategies are implemented.^41^^,^^42^ First responders and risk managers not only need to be trained on what a change in global energy supply will imply, first, the emergence of new risks linked with a change of energy supply. This has already been investigated, to some extent, in the World Economic Forum^34^ and the United Nations Office for Disaster Risk Reduction.^27^ Second, it also suggests that changes in energy systems will inevitably impact how prevention, intervention, and recovery are currently managed.

It is in this new opening energy paradigm that human systems, first responders, decision-makers and risk managers may ask themselves how risks are to be managed in the future. Future risk management strategies need to address the limits of the global energy system and its variability. The change in global energy supply, for example, calls for questions to be asked about the resilience of supply chains of anything remotely related to risk management strategies (medical, rescue equipment, …). These supply chains may be also now globalized and may face not only a change in environmental conditions but also potential changes in energy supply. This approach must be taken in conjunction with future challenges, in particular by incorporating changes to physical hazards in a climate change context. Ultimately, in such a context, it will be discussed in this article that risk management strategies depend on the environment and energetic conditions and their respective changes. To tackle these challenges there is an opportunity to support risk managers and decision makers on the systemic limits of the global energy system.

## Energy challenges

**Figure 1:**
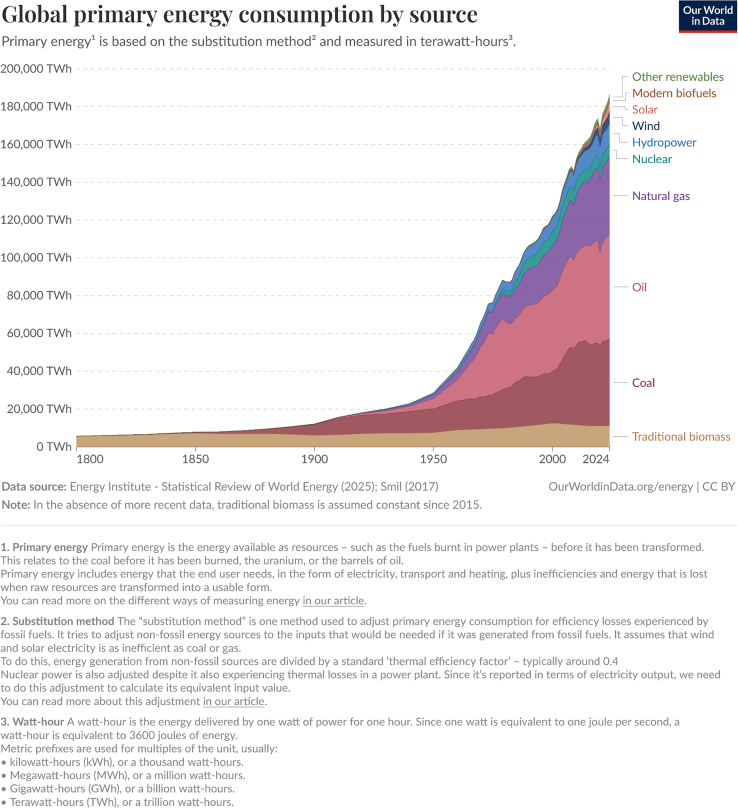
World energy consumption between 1860 and 2019 (Ritchie et al., 2020^45^) In the Y axis: Million tons oil equivalent (Mtoe), in the X axis: years. Data from Energy Institute - Statistical Review of World Energy (2025)^46^ and Smil (2017)^3^.

Whatever the system, be it an individual, a network, a city, or a nation, a system needs an increasing and constant supply of energy to grow. Humanity has relied on different sources of energy and machinery to sustain itself and a continued prosperity. However, the nature of energy and machines used by human systems changed over time.^3^ Before the industrial revolution, wind, water, or food resources (plants and animals) were the first sources of energy used; at that time, the world was entirely running on renewable energy. The exploitation of fossil resources at the beginning of the industrial era radically changed the situation, allowing humanity to progressively have access to very large quantities of energy, which was controllable and relatively easy to access. Over the last 200 years, the growing access to fossil fuels has favored the development and democratization of mechanization in all sectors of activity. This has ultimately led to an acceleration and increase in the production of goods and services in human systems, a development of cities, networks, and artificial lands.^29^^,^^43^^,^^44^ The explosion of production chains with processing sites for a given product scattered around the globe are also part of the changes that have been made possible by the increased availability of energy and the mechanization of systems. Figure 1 indicates the evolution of energy consumption between 1860 and 2019.

In 2019, fossil fuels represented more than 80% of the energy consumed by human systems^45^^,^^46^^,^^47^. Traditional biomass was one of the first carbon-based energy used by humanity. It was followed by coal, then oil and gas. Compared to oil, coal, and gas, traditional biomass (wood, peat, and so forth) is scientifically considered as a renewable energy, as it takes 20 to 30 years to grow a tree. Wood, as a primary energy source, is included as a source of CO_2_ emissions. The remaining sections are made up of hydroelectricity and nuclear power. Other New Renewable Energies (NRE) represent a smaller portion of energy consumption for humanity. The NRE represented in Figure 1 includes solar panels, wind turbines, modern biofuels, hydrogen, and other renewables (e.g., geothermal energy or tidal waves).

As Figure 1 highlights, the demand for energy has been increasing since the end of the 19th century and more abruptly after the Second World War. An interesting phenomenon to note on Figure 1 is that, as the energy consumption is increasing, new renewable energies are currently not replacing fossil fuels; they are, at the moment, filling in the growing global energy demand. All growth, whether economic growth, the development of new industries and services, urban development in developed and developing countries or the increase in the world’s population, mechanically requires an increase in energy supply.^29^^,^^48^ Additionally, Figure 1 not only indicates the energy consumption level, but it also shows a level of dependence. Indeed, the increase in the volume of energy consumption seen in Figure 1 simultaneously indicates an increase in the level of dependence of our systems on a volume of energy. Without the maintenance of a certain level of energy necessary for its functioning, human systems are subject to potential tensions in carrying out these activities. Ultimately, any dependency also indicates a level of vulnerability. They are two sides of the same coin. This heavy dependence on energy makes our systems vulnerable to a constant supply of energy to maintain their current structure and to operate.

However, the current energy supply is now facing major structural changes both in terms of available volume and energy source^4^^,^^18^^,^^49^ and therefore, future energy consumption is projected to change, leading to structural changes to current systems. Knowing the limits of current energy production and consumption should be a priority for risk managers since their risk management strategy is based on (and restricted by) the energy system in which they are implemented. Therefore, a change or degradation in energy availability represents a major systemic risk not only for human systems but also threatens current risk management strategies that are highly dependent on energy availability and its variations.

## Understanding the structural limits in the future energy supplies

### Structural limits and future scenarios for fossil fuels

**Figure 2:**
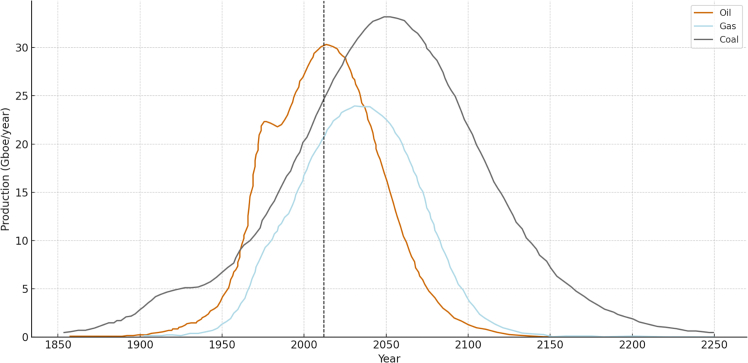
Comparison of fossil fuel production forecasts The figure has been reproduced and adapted from Maggio & Cacciola, 2012.^54^ This figure shows the production scenarios for the three main fossil fuels, oil in brown, coal in gray and natural gas in pale blue. The years 1850–2012 are based on data (2012 is indicated with a dashed line). All data represented for the period 2012–2250 is modeled. For more detailed information on the modeling please refer to Maggio & Cacciola, 2012^54^ and future updates. For these three fossil fuels, the production peaks for each of them are expected to occur in the 21st century.

Energy transitions are not merely technological challenges; they are systemic shifts constrained by physical, political, and logistical limits that shape the feasibility and pace of decarbonisation.^3^^,^^18^ Fossil fuels currently provide the majority of the global energy supply, but their availability is finite, and their extraction is increasingly constrained by geological, economic, and geopolitical factors. The concept of peak oil, first proposed by Hubbert^50^ in 1956, suggests that the extraction of finite fossil resources will inevitably reach a maximum point before declining due to the depletion of accessible reserves. At the temporal scale of humanity, the initial volumes of fossil fuels cannot be naturally recreated as it took several million years of time as well as complex geological processes to constitute them. Early projections by Campbell and Laherrère^51^ estimated a peak in oil production by the early 21st century; however, advances in extraction technologies, especially unconventional methods such as shale oil and gas, have delayed the anticipated peak.^52^ These new extraction methods underscore the adaptability of energy systems, but they also highlight the inevitable decline of easily accessible fossil fuels over time^53^ Analyses have long anticipated a peaking and eventual decline in fossil fuel production. Maggio and Cacciola,^54^ for instance, estimate the production peaks for oil, coal, and natural gas based on the state of known reserves, modeling production trends from 1850 to 2250. Their work, shown in Figure 2, distinguishes between the historical record (1850–2012) and projected future output, reflecting a general consensus that peak production will occur for all three fossil energy sources. Although exact timing and volumes remain uncertain^55^^,^^56^^,^^57^ the long-term trajectory is clear: the extraction of fossil fuels at current volumes cannot continue indefinitely, especially alongside growing global energy demand.

**Figure 3:**
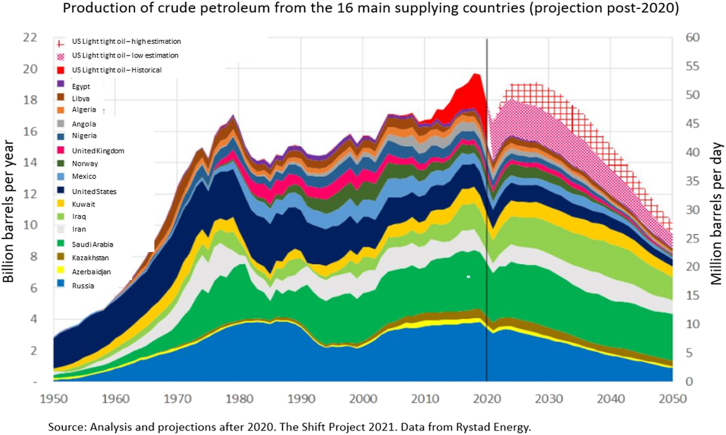
Crude oil production of the 16 main supplier countries for Europe (post 2020 projections) Sources: The Shift Project (2021)^49^ Data from Rystad Energy data. Translated from French.

This broader trend is also reflected in more recent analyses. Figure 3, derived from data by The Shift Project^49^ and Rystad Energy, details projected declines in oil production from the top 16 supplier countries for Europe. Figure 3 highlights the anticipated gradual decline driven by resource depletion and reduced investments, especially following the 2020 downturn due to COVID-19. Even if the world does not “run out” of fossil fuels, a decline in their accessibility and affordability, coupled with high global dependency and global competition, can produce profound economic and social disruptions. For risk managers and first responders, such trends highlight the importance of anticipating supply shocks and their cascading economic or operational consequences across critical infrastructure, emergency services, or public health systems. Peak oil therefore represents more than just a physical constraint on energy production or an unclear concept. As well as indicating a high level of dependence on fossil fuels, it marks a structural turning point, requiring the reconfiguration of energy systems, consumption patterns, and resilience strategies. A shift toward a more diversified, flexible, and lower-carbon energy future is essential, and increasingly urgent.^58^

**Figure 4:**
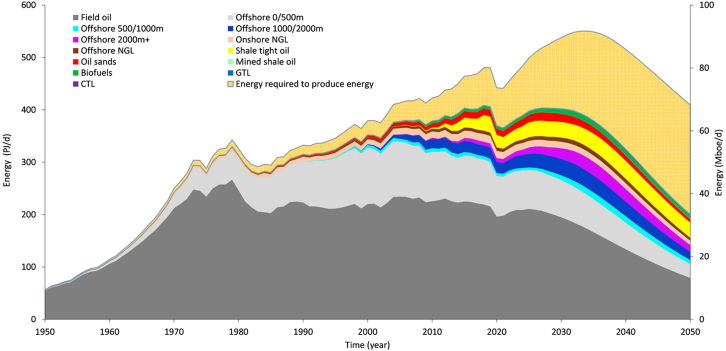
Average oil liquids net-energy production from 1950 to 2050, compared to the gross energy Delannoy et al., 2021.^18^

More recently, Delannoy et al.^18^ discusses the possibility of a low-carbon energy transition in the context of the depletion of non-renewable fossil fuels after a possible peak of these resources. Delannoy et al.^18^ explore the possibilities associated with fossil fuel decline by outlining two main scenarios. The first type of scenarios involves a rapid transition toward low-carbon energy, leveraging renewable technologies and electrification. Technically feasible within a short time frame, this scenario would use only a modest share of remaining fossil fuels. However, it would require an unprecedented rate of deployment, global governance alignment, and access to large quantities of critical materials such as lithium, copper, and rare earth elements.^29^^,^^59^ Moreover, these resources are unevenly distributed, creating new geopolitical tensions and dependencies. Compounding these challenges is the declining Energy return on investment (EROI).^18^^,^^60^ As EROI declines, so too does the net energy surplus available to power economies and human systems, making transitions costlier and more complex. The second scenario assumes a delayed transition, where fossil fuel use continues until resource constraints force an abrupt and unmanaged shift. In this context, rising energy costs, geopolitical instability, and deteriorating infrastructure could exacerbate socioeconomic inequalities and undermine commitments to climate targets as defined in IPCC.^20^ As Garrett et al.^12^ show, global energy use remains tightly coupled to GDP, suggesting that declining energy availability could suppress economic output and limit the financial capacity for transition investment. Additionally, the inertia of existing institutions and infrastructure lock-in may further slow adaptation, compounding systemic risks.^61^ Figure 4, from Delannoy et al.^18^ illustrates projected oil production declines by 2050, emphasizing the strategic challenge: can renewable systems be deployed fast enough to fill the energy gap left by fossil fuels? The answer remains uncertain. What is clear, however, is that the longer societies delay action, the narrower the window becomes for a managed transition that avoids cascading systemic shocks. An uncoordinated decline in fossil fuel supply may generate tensions across political, industrial, economic, and social systems.^62^ Critically, it can also disrupt supply chains and hinder the production of essential goods, thereby compromising the operational capacity and management effectiveness of first responders and risk managers. To address these challenges, it is essential to integrate fossil fuel vulnerability into risk assessments and emergency planning. By anticipating variable energy availability and potential infrastructure stress points, organizations can better prepare for complex crises, safeguard essential services, and design adaptive, future-ready resilience strategies grounded in an understanding of structural energy limits.

#### Political factors and climate agreements influencing global energy consumption and fossil fuel reduction

**Figure 5:**
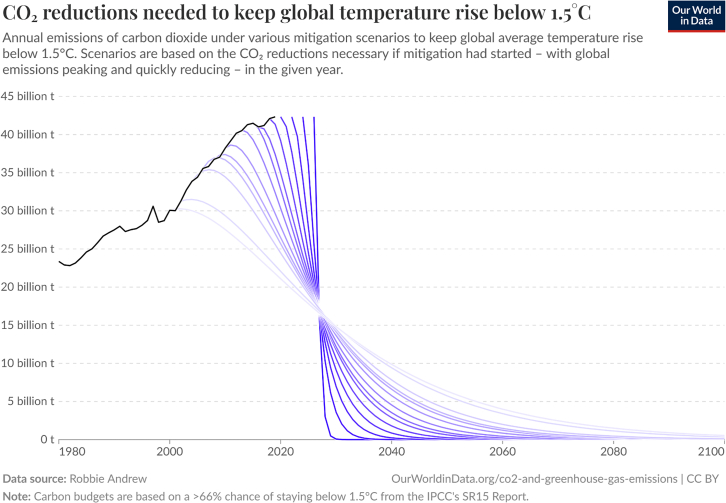
CO_2_ mitigation curves for 1.5°C Source: Robbie Andrew, processed by Our World in Data.^63^ “CO2 mitigation curves to meet a 1.5C target”. Based on carbon budgets from the IPCC Sixth Assessment Report (2021), adjusted for emissions in 2020–21. Also available in Hickel & Kallis (2019).^64^

Global energy consumption patterns are heavily shaped by political dynamics. To address climate change associated with energy use, the IPCC^20^ emphasizes the critical importance of reducing reliance on fossil fuels, particularly through government action. The Paris Agreement, established at COP21 in 2015, requires nations to cut greenhouse gas emissions by limiting fossil fuel consumption in human activities, with the aim of keeping the global temperature rise well below 1.5°C. Figure 5 below shows the different curves for reducing greenhouse gas emissions to stay below 1.5°C.

While international agreements provide a framework for coordinated climate commitments, the implementation of these targets, particularly Nationally Determined Contributions (NDCs), which are country-specific targets for reducing greenhouse gas emissions, introduces political and economic risks. Even if it is a necessity to avoid the effects of climate change, moving away from fossil fuel industries may destabilize economies dependent on resource extraction, triggering resistance and policy volatility.^18^^,^^58^^,^^61^ The feasibility of energy transition scenarios is therefore shaped not only by technological or environmental factors but also by political alignment and geopolitical stability. Further complicating matters, the renewable transition depends on access to critical minerals such as lithium, cobalt, and rare earth elements (discussed in section renewables energies and their limits). These are often concentrated in a few countries, creating new geopolitical dependencies that may hinder progress toward COP targets and increase vulnerability to international tensions. The International Renewable Energy Agency^65^ (IRENA) has highlighted how the geographic concentration of key minerals can endanger global supply chains during times of crisis. However, political frameworks can also accelerate the transition.^66^ Supportive policies, such as renewable energy subsidies, tax incentives, and green investment programs, encourage both public and private sectors to adopt clean technologies.^67^ Moreover, well-designed climate and energy policies can enhance the resilience and flexibility of supply chains, providing critical co-benefits for first responders and risk managers who depend on stable access to infrastructure and essential goods during crises.^66^^,^^68^

In political instability, sudden policy shifts, or international conflict can disrupt supply chains, delay infrastructure development, and reduce energy security. As Wang et al.^69^ note, in countries with high dependence on fossil fuel rents, financial and governance risks may derail transition plans or reduce their credibility. For first responders and risk managers, these political dynamics are not abstract; they directly influence the availability and reliability of energy needed to operate emergency services and maintain critical infrastructure. Energy disruptions linked to political factors can intensify the impacts of natural hazards or delay response and recovery efforts.^64^^,^^69^ To build resilient systems, emergency planners must integrate political risk assessments into preparedness strategies. This includes accounting for potential disruptions in energy availability due to conflict, sanctions, or supply bottlenecks. A proactive approach that considers both the vulnerabilities and opportunities created by the energy transition can help ensure the continuity of emergency services and protect communities during future crises.

### Renewables energies and their limits

The potential depletion of fossil fuel resources is not the only factor calling into question the long-term sustainability of current energy systems. Maintaining today’s levels of energy consumption will require not only alternative sources of energy but also profound structural changes in how human systems operate. Among different scenarios, renewable energies (NRE) and the electrification of human systems are often discussed as the main alternative to fossil fuels to supply the energy demand with low greenhouse gas emissions.^70^ However, replacing fossil-based energy with renewables induces profound systemic changes and reveals a series of structural, material, and geopolitical constraints.

#### Electricity as final energy: What it means for the transition

**Figure 6:**
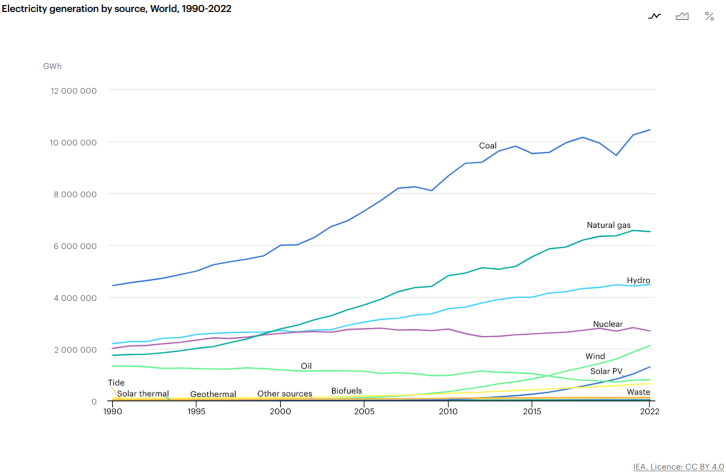
Electricity generation by source, world, 1990–2022 Source: International Energy Agency (IEA), reproduced under the terms of the Creative Commons CC BY 4.0 license. IEA, 2025.^47^

Electricity is a final energy form, produced by transforming primary energy sources—most commonly gas, coal, and oil. Unlike fuels such as coal or oil that can be directly combusted, electricity must first be generated before it can be used. In 2022, fossil fuels continued to dominate global electricity generation: coal accounted for approximately 10,450 TWh, natural gas for around 6,520 TWh, and oil for about 800 TWh^47^, Figure 6 illustrates the distribution of energy sources used in electricity production, underscoring the continued reliance on fossil fuels worldwide.

Electrification is a key pillar of energy transition, often promoted to reduce direct emissions in sectors such as transport, heating, and industry. However, scaling electricity systems to meet such expanded roles globally involves major infrastructure development in generation, distribution, and especially storage. The intermittency of renewables, such as wind and solar, introduces complexity, as electricity must be available on demand, even when natural conditions fluctuate.^4^ Unlike fossil fuels, which store energy inherently, renewable-based electricity requires parallel storage infrastructure to maintain reliability. For risk managers, this dependency introduces a new layer of exposure. A disruption in electricity generation or distribution, due to fuel shortages, infrastructure failure, or natural hazards, can cascade into a wide range of failures within first responders and risk management strategies, including communication, emergency response, or water systems for example.^16^

#### Mineral dependencies and the challenge of scaling

Most renewable energy technologies are highly mineral and material intensive. Wind turbines, photovoltaic panels, electric vehicles (EVs), and stationary battery systems all depend on a suite of critical minerals such as copper, lithium, cobalt, nickel, and rare earth elements. These materials are both finite and geographically concentrated, posing significant challenges for global supply chains and energy transition timelines. Ultimately, the global energy transition involves a fundamental shift, requiring a move from dependence on fossil fuels to increased reliance on critical minerals, which introduces new vulnerabilities related to resource availability, supply chain complexity, and geopolitical concentration.^71^

**Figure 7:**
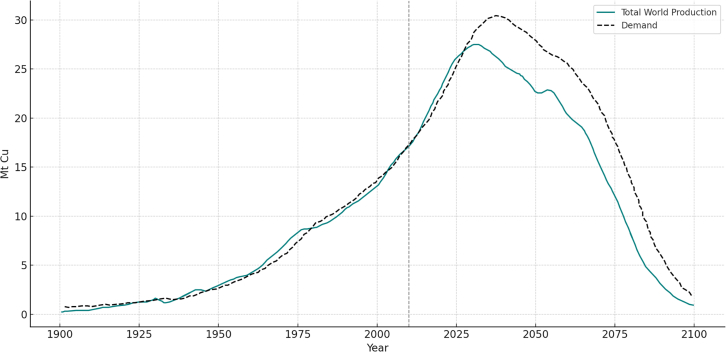
Global Cu production (in Mt Cu) as modeled by GeRS-DeMo in dynamic demand mode The years 1900–2014 based on data (2014 is indicated with a dashed line). All data represented for the period 2014–2100 is modeled. The figure has been reproduced and adapted from Northey et al., 2014^72^ Data courtesy of Dr Stephen Northey.

As an example, Figure 7 illustrates copper production trends from 1900 to 2100. The role of copper in the energy transition is major, a foundational component in electrification infrastructure, extensively used in power grids, EV charging stations, wind turbines, and solar photovoltaic systems. The left-hand section shows historical copper output from major producing countries, while the right-hand section presents modeled projections. According to Northey et al.^72^ copper production is expected to continue rising and reach a global extraction peak around 2040, followed by a gradual decline through the end of the century. This pattern mirrors the dynamics observed in fossil fuel extraction: as high-grade ores become depleted, production slows despite rising demand, creating systemic bottlenecks that could constrain renewable energy deployment.

**Figure 8:**
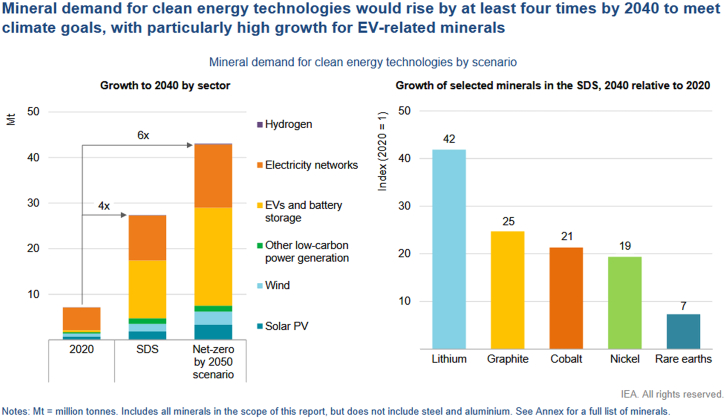
Projected growth in mineral demand for clean energy technologies Mineral demand is expected to increase by at least 4-fold by 2040 to align with global climate goals, with particularly sharp growth in minerals used for electric vehicles (EVs). Source: International Energy Agency (IEA), *The Role of Critical Minerals in Clean Energy Transitions*, 2022.^59^ Reproduced under the terms of the Creative Commons CC BY 4.0 license.

Beyond copper, other minerals central to clean energy systems face significant demand pressures. Figure 8, from IEA^59^ presents projected global demand for key minerals under two climate policy scenarios: the Sustainable Development Scenario (SDS) and the Net-Zero by 2050 scenario. Under SDS, mineral demand is projected to quadruple by 2040; under the more ambitious Net-Zero pathway, it could increase 6-fold. Lithium, cobalt, nickel, and graphite, crucial for battery production, are expected to see the most dramatic growth, driven by their central role in EVs, storage technologies, and renewable energy deployment.

**Figure 9:**
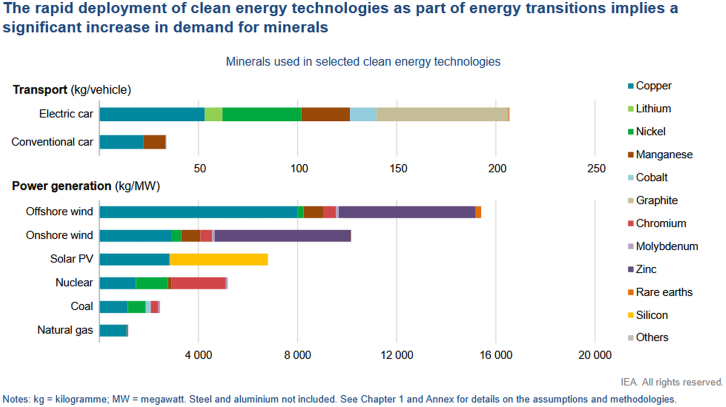
Mineral use in selected clean energy technologies This figure compares the average mineral requirements (in kilograms per megawatt) for various clean energy technologies. Steel and aluminum are not included. Abbreviations: kg = kilogram; MW = megawatt. Source: International Energy Agency (IEA), The Role of Critical Minerals in Clean Energy Transitions, 2022^59^ Reproduced under the terms of the Creative Commons CC BY 4.0 license.

This rising demand is not confined to the energy sector. These same minerals are in high demand in telecommunications, defense, semiconductors, transport, and medical devices, creating a multi-sectoral competition for limited and slow-to-scale resources. As shown in Figure 9, the mineral intensity of an electric car is several times higher than that of a conventional internal combustion vehicle, and wind or solar power installations require far more minerals per megawatt (MW) than coal or gas plants. For example, offshore wind uses around 15 times more mineral resources per MW than a gas-fired plant, and solar PV requires up to 6 times more.^59^

Furthermore, future competition may arise not only between sectors, but also within the energy system itself, between the machines that generate electricity (e.g., wind turbines and solar farms) and those that consume it (e.g., EVs, heat pumps, and data centers). This internal conflict can create allocation dilemmas, especially during crisis periods when strategic decisions must be made about prioritizing power to critical infrastructure versus economic or social uses. From a systems perspective, these dynamics pose a profound challenge to scaling renewables globally and equitably. High material requirements mean that the pace of the energy transition is increasingly dependent on mineral exploration, investment, and refining capacity, which lag significantly behind projected needs. In addition, environmental and social impacts of mining, including water use, land degradation, waste, and labor rights, can trigger resistance in both producer and consumer countries, increasing risk factors.^73^

For first responders, disaster risk managers, and critical infrastructure planners, dependencies on critical minerals introduce several strategic vulnerabilities. Rising global competition for minerals such as lithium, cobalt, and copper may lead to increased costs for emergency technologies and operational tools that rely on these resources, such as electric vehicles, communication systems, or mobile storage units. In parallel, material shortages can delay the deployment of decentralized energy solutions, including microgrids and backup systems, that are crucial in crisis contexts. These constraints reduce the resilience of infrastructure systems in the face of cascading disruptions, particularly when energy supply chains are already stressed. Furthermore, such dependencies pose a challenge to energy sovereignty, particularly in regions reliant on imported equipment or materials, potentially limiting autonomy during emergency responses and recovery operations.

#### Nuclear and hydrogen: Opportunities and constraints

While renewables face intermittency and mineral dependency, other energy sources, such as nuclear and hydrogen, offer complementary strengths and are often presented as solutions to sustain high levels of energy availability within human systems. However, these options also entail significant challenges. This section aims to move beyond overly optimistic reliance on alternative energy sources such as nuclear energy and hydrogen as silver bullet solutions, emphasizing instead the need to integrate them thoughtfully within a broader, systemic energy strategy. Nuclear power is a dense, “low-carbon” energy source capable of continuous electricity generation.^20^ However, it is a final energy requiring complex systems to operate safely.^74^ At the moment, nuclear plants are costly, slow to build, reliant on uranium, and vulnerable in both conflict and disaster contexts. The 2022 Russian occupation of the Zaporijia plant in Ukraine^75^ and the Fukushima Daiichi accident in Japan^76^ illustrate the strategic and cascading risks associated with nuclear infrastructure. Furthermore, the challenge of long-term radioactive waste management remains complex and costly.

Hydrogen, particularly green hydrogen produced via electrolysis powered by renewables, is a promising vector for decarbonizing heavy transport and industry. However, to produce hydrogen in sufficient quantities to replace the volumes of oil consumed, it is necessary to create power plants upstream of the hydrogen production chain to create the volume of hydrogen required.^77^ Its production requires substantial energy inputs. As of 2022, global hydrogen production from renewable sources accounted for less than 1% of total production and remains 2–3 times more expensive than fossil alternatives.^77^^,^^78^^,^^79^ Scaling hydrogen requires vast new infrastructure, including electrolyzer factories, transport pipelines, and storage, each with material, energy, and governance implications. As McDowall and Eames^80^ argue, hydrogen is more likely to coexist with other energy carriers than to replace fossil fuels entirely. Strategic decisions must be made about whether limited electricity supplies should support hydrogen production or serve core societal needs.

Neither nuclear nor hydrogen is a one-size-fits-all solution. Their deployment must be embedded within broader systems-level planning, integrated with renewable capacity, grid flexibility, and long-term risk reduction strategies. Nuclear power may suit countries with stable governance, low seismic risk, and robust safety institutions, while hydrogen may prove essential in regions with abundant renewables and high industrial energy demands. For risk managers and first responders, this has two key implications. First, expectations about the role of hydrogen and nuclear energy must be grounded in realistic assessments of regional capability, infrastructural maturity, and vulnerability. Second, both technologies must be embedded within broader resilience strategies that address long-term infrastructure risk, governance gaps, and evolving climate threats. First responders and risk managers must engage in discussions with decision-makers about these changes, as the restructuring of energy supply and systems alters the nature of risk and vulnerability within these systems, particularly in the context of climate change, where climate-related extreme events are set to change.

#### Advancements in integrated energy systems: Strengthening resilience for risk management

Integrated energy systems are reshaping how energy is produced, stored, and distributed, enhancing resilience against climate threats and system vulnerabilities. Hybrid systems, such as cogeneration, district heating and cooling, and combined heat and power (CHP) optimize energy use and reduce waste, providing greater reliability during extreme events.^81^^,^^82^

Smart grids have significantly improved energy system performance by enabling real-time monitoring, advanced metering, and communication networks. These features facilitate rapid responses to demand fluctuations and infrastructure disruptions, improving the reliability of energy supply during crises.^83^ Microgrids offer an added layer of resilience by allowing localized generation and storage that can operate independently from the main grid. This is particularly crucial in disaster-prone or remote areas, where energy continuity is essential for first responders and healthcare facilities.^84^^,^^85^ The role of smart microgrids in supporting emergency services. For instance, Kwasinski et al.^85^ demonstrated how microgrids maintained power for critical services following Hurricane Katrina, while Hamidieh and Ghassemi^86^ emphasized their utility in ensuring continuity for public safety operations. Such systems enable first responders to function effectively when the central infrastructure is compromised. To address the inherent variability of renewables, innovations in energy storage, such as lithium-ion and solid-state batteries, as well as thermal storage, have proven essential. These technologies store excess energy during high production periods and release it as needed, thus enhancing stability and grid reliability.^87^^,^^88^ For example, molten salt thermal storage extends the usability of solar power into periods of peak demand or grid failure. Integrating renewable energy with storage systems and flexible infrastructure, such as hydrogen electrolysis or power-to-heat systems, supports both decarbonization and system resilience.^89^ Co-locating generation with storage or using technologies such as heat pumps enables sector coupling and reduces dependence on fossil fuels.

For risk managers and DRR professionals, these systems are vital across all phases of crisis response: ensuring energy availability during initial impacts, offering modular backup solutions when primary systems fail, and supporting decentralized recovery. As Guerrero et al.^82^ and Lund et al.^83^ note, the scalability and modularity of these systems make them particularly suitable for post-disaster recovery and future-proofing critical infrastructure. Nonetheless, while promising, these technologies must be viewed within the broader context of an uncertain energy transition. Their success depends on their integration within existing systems, policy coherence, and regional adaptability.^3^^,^^90^ As climate and geopolitical risks evolve, first responders and planners must adopt flexible, systemic approaches that acknowledge ongoing energy volatility and infrastructure fragility.

### Climate risks to energy systems: Cascading impacts and operational vulnerabilities

#### The effect of climate change on energy systems

In addition to the variability and limitations of energy sources, climate change and environmental degradation significantly affect the performance, stability, and reliability of global energy systems. These impacts extend across all stages of the energy chain, production, distribution, storage, and consumption, creating new stressors and amplifying existing vulnerabilities. Table 1, from Schaeffer et al.^91^ presents an overview of these interactions, mapping the exposure of various energy sources and sectors to different types of climate hazards and extreme weather events. Both long-term trends (e.g., rising average temperatures) and acute events (e.g., hurricanes, floods) can destabilize energy infrastructure, reduce efficiency, and compromise continuity of supply. The first column describes the type of energy sector, while the second column lists possible climate variations and extreme events. The third column details the related impacts on these energy sectors.

**Table 1:** Summary of climate change impacts on energy systems Schaeffer et al. (2012)^91^

Energy sector	Climate variables	Related impacts
Thermoelectric power generation (natural gas, coal, and nuclear)	Air/water temperature; Air/water temperature, wind, and humidity; Extreme weather events	Cooling water quantity and quality, cooling efficiency, and turbine operational efficiency
Oil and gas	Extreme weather events: Air/water temperature, flooding.	Erosion in surface mining, disruptions of offshore extraction, disruptions of onshore extraction, disruptions of production transfer and transport, disruption of import operations, downing of refineries, cooling water quantity and quality in oil refineries
Biomass	Air temperature, precipitation, humidity; Extreme weather events, Carbon dioxide levels	Availability and distribution of land with suitable edaphoclimatic conditions (agricultural zoning); Desertification, Bioenergy crop yield
Hydropower	Air temperature, precipitation, and extreme weather events	Total and seasonal water availability (inflow to plant’s reservoirs), Dry spells, Changes in hydropower system operation
Demand	Air temperature, precipitation	Evaporation from reservoirs, increase in demand for air conditioning during the summer, decrease in demand for warming during the winter, and increase in energy demand for irrigation
Wind Power	Wind and extreme weather events	Changes in wind resource (intensity and duration), changes in wind shear, and damage from extreme weather
Solar Energy	Air temperature, humidity, and precipitation	Insolation changes (cloud formation), decrease in efficiency due to decrease in radiation, decrease in efficiency due to ambient conditions
Geothermal	Air/water temperature	Cooling efficiency
Wave Energy	Wind and extreme weather events	Changes in wave resource

The energy sector, already transitioning to integrate renewable sources, faces a feedback loop: energy consumption patterns contribute to climate change, which in turn threatens the stability and resilience of current and future energy systems. Recent studies highlight a variety of ways in which climate change disrupts energy systems, from extreme weather events that threaten renewable energy generation to rising temperatures that affect grid efficiency and increase overall energy demand.^91^^,^^92^ For instance, heatwaves reduce the output of solar panels and increase cooling demand, straining transmission networks.^93^ Storms and floods can damage wind turbines and solar farms, limit electricity generation and increasing maintenance costs.^93^ Research by Mukherjee and Nateghi^94^ also highlights that climate change is expected to increase the frequency and intensity of these extreme weather events, necessitating that first responders consider the implications for energy stability and availability.

This evolution presents critical planning challenges for risk managers, who must now account for more frequent climate shocks and their interactions with aging or stressed energy infrastructure. The compounding nature of these hazards demands multi-layered, flexible risk management strategies that anticipate cascading effects, such as simultaneous damage to both generation and transmission assets, and incorporate diversified backup systems.

Beyond episodic weather extremes, climate variability introduces longer-term challenges for energy system stability. Fluctuations in temperature, precipitation, and seasonal weather patterns will increasingly disrupt energy planning and infrastructure performance. These effects are particularly pronounced for renewables, where output is often weather-dependent. For example, droughts can severely impact hydropower availability, while cloud cover and changing wind patterns affect solar and wind energy outputs. As noted by Cronin et al.^95^ these fluctuations create greater unpredictability and complicate supply demand balancing. These dynamics are compounded by chronic stressors. Bull et al.^96^ and Schaeffer et al.^91^ show that rising temperatures can reduce transmission efficiency, requiring more energy to deliver the same output and limiting grid capacity during peak demand. This challenge is especially significant during heatwaves, when the need for electricity surges while grid performance declines. Cronin et al.^95^ emphasize that such thermal stress on transmission lines can increase the risk of blackouts in vulnerable regions. To address these risks, adaptation measures such as upgrading transmission lines, integrating temperature-resistant materials, and redesigning cooling systems are essential. Climate change also exacerbates pre-existing physical vulnerabilities in global energy infrastructure. For instance, higher temperatures reduce the efficiency of fossil, nuclear, and solar thermal plants, while flooding can submerge substations and damage gas pipelines. Infrastructure that was designed under historical climate norms may no longer be sufficient to withstand future extremes.

Importantly, climate change not only affects physical infrastructure, but it also threatens energy supply chains and strategic reserves. Rising global demand for electricity, driven by electrification and economic growth, combined with climate-induced disruptions, may outpace existing system capacities. The Energies^8^ report stresses that mapping energy risks across systems reveals new points of systemic fragility, while KPMG^7^ highlights the need for enhanced systemic risk assessments that consider cascading and cross-sectoral failures. For risk managers, integrating these insights into scenario planning, vulnerability assessments, and infrastructure design is no longer optional. It is a fundamental component of disaster risk reduction and long-term resilience. Anticipating the multifaceted effects of climate change, from thermal stress and weather extremes to supply chain vulnerabilities, will allow decision-makers to prepare for disruptions, sustain essential services, and reduce recovery time after crises. In summary, the impacts of climate change on energy systems are profound, accelerating, and increasingly systemic. First responders and disaster risk managers must adopt proactive strategies that integrate energy system vulnerabilities into their preparedness frameworks. This includes not only responding to immediate threats, but also anticipating long-term shifts in infrastructure performance and supply chain reliability. Thus, building adaptive and climate-resilient energy systems is central to safeguarding critical services, ensuring operational continuity, and protecting the communities that depend on them.

#### The effect of climate change on mining activities: Minerals as a dependency for the energy systems of the future

The production of minerals essential for the energy transition is also increasingly vulnerable to the impacts of climate change. Odell et al.^97^ note that mining operations, which often rely on stable environmental conditions, are generally unprepared for climatic variations. These conditions are crucial for the different phases of mining, including exploration, extraction, and transportation. Pearce et al.,^98^ and Odell et al.^97^ highlight that climate change is already affecting mining sites through issues such as water supply shortages, the destruction of equipment during extreme events, and reduced working hours due to heatwaves. Most mining-related infrastructure does not account for these climatic fluctuations, exposing operations to higher risks and uncertainties as climate change accelerates. This underscores a fundamental limitation for the role of minerals, metals, and rare earths in the energy transition, as climate change threatens both current and future energy systems.

**Figure 10:**
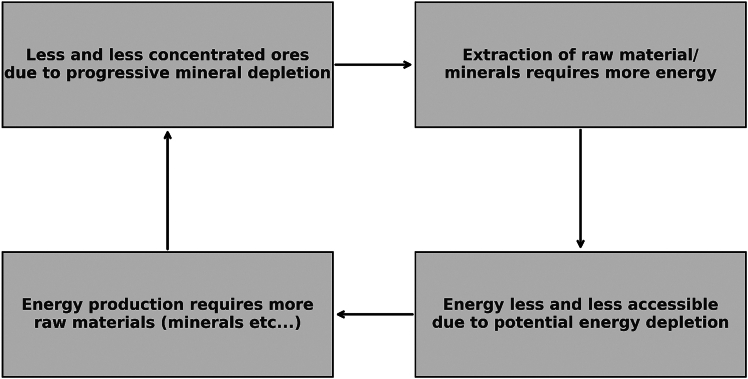
Relationships between the energy and mineral sector in a growing system Source: Sketch drawn according Bihouix, 2020.^99^

As human systems continue to evolve, there is a shift from a reliance on fossil fuels to a growing dependence on mineral resources essential for renewable energy technologies. However, as previously stated, the energy transition introduces a new set of constraints, as both fossil and mineral resources are subject to geological, physical, and geographical limitations. The capacity to sustain current growth within human systems, with an expectation of limitless energy generation from renewable sources, is likely limited by these resource dependencies. The increasing demand for minerals, against a backdrop of finite resources and environmental constraints, presents a systemic challenge that affects multiple time scales.^70^ This interdependence between energy and mineral resources complicates the pathway toward a sustainable energy future. While renewable technologies are essential for reducing carbon emissions, the raw materials required for these technologies are subject to scarcity, supply chain vulnerabilities, political context and environmental impacts.^18^ In addition, the current narrative around “green growth” often overlooks these challenges, suggesting that renewable energy alone can sustain the growing demands of human systems without considering the long-term viability of the resources involved.^70^ This relationship between energy and minerals can be visualized in Figure 10, which illustrates the cyclical dependencies and limitations inherent in transitioning to renewable energy systems.

In summary, as climate change disrupts both fossil fuel and mineral supply chains, the effects extend beyond the current energy system to threaten, to some extent, the feasibility of future renewable-based systems. This creates a feedback loop: current energy consumption patterns drive climate change, which then impacts the resource availability needed for sustainable energy. Moreover, as Delannoy^18^ notes, the availability of key minerals required to transition the entire energy system is limited by geological and economic constraints, raising questions about the feasibility of large-scale renewable deployment and underscoring the challenges of sustaining “green growth” in a context of finite resources.

#### Implications for risk managers

While energy supply and demand management is primarily handled at national or regional levels, shifts in global energy availability can have substantial effects locally, influencing national energy costs, supply chain stability, and the effectiveness of risk management.^5^^,^^6^ For first responders, DRR professionals, and decision-makers, understanding the dynamics outlined by Delannoy et al.,^18^ is essential for effective planning and resilience-building in a context of energy transition and climate change. As Delannoy et al.,^18^ indicates, the window for transitioning to low-carbon energy systems is limited by both resource constraints and geopolitical factors. The potential for both rapid or slow transition scenarios poses unique risks, including fluctuating energy prices, resource scarcity, and heightened competition for remaining fossil fuels. In a context where energy could become increasingly scarce or expensive, first responders may face growing constraints in deploying the full range of prevention, intervention, and recovery strategies at the necessary scale, which could limit their effectiveness in responding to extreme events. Reduced energy availability could limit critical recovery operations, impact the resilience of emergency response systems, and make it increasingly challenging to maintain existing DRM strategies. Recognizing these risks allows risk managers to integrate energy transition scenarios into their strategic planning, anticipate systemic vulnerabilities, and adapt DRM frameworks to the dual pressures of an evolving energy landscape and accelerating climate change. In doing so, they can better prepare for disruptions in energy supply chains, mitigate the impact of rising costs on critical infrastructure, and support more resilient and sustainable development pathways aligned with broader societal objectives.

Additionally, the energy transition introduces several technological limitations for risk management equipment, particularly in fields such as emergency response, where reliable, high-energy-density power sources are critical. For example, electric-powered emergency vehicles, such as fire trucks or ambulances, may face challenges in achieving the range and reliability needed for uninterrupted response operations, especially in remote or disaster-prone areas where charging infrastructure may be limited.^100^ Furthermore, while battery technology continues to improve, current energy storage solutions may not yet support extended-duration operations for equipment such as portable generators, drones, and other devices essential in emergency response and monitoring.^87^ Additionally, renewable energy sources such as solar or wind are inherently intermittent, which poses challenges for ensuring continuous power for vital equipment during prolonged or intense disaster scenarios.^101^ These technological constraints highlight the need for research into energy-dense, reliable power solutions that can support risk management strategies effectively during the energy transition and operations around extreme events.

#### Understand the potential limits of change in the global energy system. A summary of potential systemic limits, vector of risks, and strategy to adopt

The global energy system is therefore undergoing significant transformations, whether by political choice or as a consequence of diminishing resources. The various limitations described in the previous sections raise questions about the uncertainties associated with maintaining the current energy system as well as current disaster risk management strategies. Decision-makers and risk managers need to consider these uncertainties and structural changes to adapt effectively. The challenges they face in this context are numerous and can be summarized as follows.

(1) The first challenge in the electrification of growing human systems and moving away from fossil fuels is the need to close existing fossil fuel power plants and replace them with plants that run solely on renewable energy to limit CO2 emissions and reduce climate change and related hazards. Electrification requires an increase in the consumption of minerals and metals to create wind turbines, solar panels, and other renewable energy technologies. This increase in mineral and metal consumption, in turn, raises energy demand (see Figure 10). Renewable power plants must produce similar electricity volumes as current thermal power plants to limit disruptions in human systems that depend on a stable level of energy supply.
(2) Electricity constitutes only a portion of the total energy consumed by human systems. Within the perspective of “green growth,” renewable energies need to match not just current electricity generation levels (as seen in Figure 6) but also the broader energy demands outlined in Figure 1. To maintain existing human systems, living standards, and to support further growth, renewable power plants must meet the energy supply levels currently provided by fossil fuels by 2050 to align with the Paris Agreement targets. Risk managers should anticipate reduced energy availability, potentially higher costs—depending on their contexts, and potential resource scarcity, impacting their strategies for disaster preparation, response, and recovery.
(3) Beyond replacing power plants, maintaining the current system requires transforming existing machinery that relies on fossil fuels to operate on electricity or other low-carbon energy sources. This challenge encompasses any vehicles, industrial equipment, and even household systems, necessitating substantial investments in minerals, technology, and research. Such a transition will likely increase demand for electricity, and risk managers must prepare for significant changes in essential tools and strategies. As human systems move toward the goals of the Paris Agreement, adaptation will be required in emergency response tools and strategies.
(4) Mineral resources, such as fossil fuels, are unevenly distributed across the globe. Geographical disparities influence trade and are further shaped by geopolitical dynamics and commercial competition. Strong competition for mineral access in a context of electrification may drive up the costs of goods, thereby impacting development across different regions^18^ Current supply chains, including those critical for risk management, are susceptible to these structural changes, with potential disruptions extending to essential infrastructure components.
(5) The combination of climate change and energy supply limits presents a particularly chaotic and unpredictable risk vector. Both phenomena contribute to global systemic changes that vary by geography, potentially triggering regional and local impacts based on each area’s specific vulnerabilities. This underscores the need for decision-makers and risk managers to assess whether their existing strategies can address such profound shifts. Today’s disaster risk management approaches are deeply dependent on fossil fuels and global supply chains. A shift toward renewable energy raises critical questions about the feasibility of replacing fossil-fueled systems with electric or hydrogen-powered alternatives and how emergency and medical equipment supply chains, dependent on oil, will adapt to the energy paradigm shift.

It is in this new paradigm of human systems that decision-makers and risk managers must ask themselves how risks are to be managed in the future? Are the strategies currently in place able to address this issue? Risk management strategies today, such as the systems in which they are implemented, are highly dependent on fossil fuels. Is it possible to change the equipment used today from fossil fuels to electricity? Will fire trucks, for example, be as efficient and able to operate in the same places if they are powered by electricity or hydrogen? The systemic change linked with energy supplies also requires questioning about the supply chains of emergency and medical equipment, which are now globalized and therefore highly dependent on oil and the current energy supply.

In the context of climate change and energy transition, risk management strategies must evolve to ensure resilience. The interconnectedness of energy systems and climate impacts demands innovative tools to anticipate, mitigate, and respond to systemic risks.^102^ Scenario analysis offers a structured method to explore possible futures, such as energy shortages, renewable integration challenges, and shifts in climate policy. By examining detailed projections of global energy futures, risk managers can prepare for disruptions and design contingency plans.^103^^,^^104^ Integrating these scenarios into planning ensures preparedness across a range of outcomes.

Vulnerability assessments provide a systematic approach to identifying weak points in energy systems, revealing critical supply chain dependencies and infrastructure exposed to climate threats such as extreme weather^101^^,^^105^. For instance, assessing transport networks or grid robustness allows targeted interventions, reducing outage risks and improving recovery times.

Adaptive risk management frameworks offer an essential tool well suited for navigating the dynamic landscape of modern energy systems. These frameworks promote flexibility through continuous monitoring and iterative assessments, enabling timely responses to changing conditions.^90^ Real-world applications, such as responses to Hurricane Sandy, illustrate their value. Following the storm’s devastation, risk managers employed scenario analysis and vulnerability assessments, leading to hardened infrastructure, microgrids, and updated emergency protocols.^106^^,^^107^ Similarly, California’s adaptive frameworks integrate climate forecasts and real-time monitoring to address wildfire risks, enabling rapid adjustments to energy distribution.^108^ Such frameworks are essential in a rapidly changing energy landscape, providing the flexibility to navigate fluctuating energy prices, supply disruptions, and unexpected environmental impacts.^90^ For risk managers, employing proactive strategies, scenario analysis, vulnerability assessments, and adaptive frameworks, is crucial to safeguarding energy systems amid climate and transition-related risks.^90^^,^^102^^,^^109^ These tools enable resilience by identifying vulnerabilities, preparing for future scenarios, and adapting strategies in real time. Such approaches are essential for maintaining reliable, sustainable energy systems under evolving threats.

## Conclusion

The transition toward a low-carbon, renewable energy-based system presents both significant opportunities and profound systemic challenges. As energy systems evolve, so too do the risks they produce and inherit. For risk managers and decision-makers, understanding and adapting to these energy dynamics is essential, not only to protect critical infrastructure but also to ensure the continuity of essential services during increasingly complex crises. This article has argued for a systemic and forward-looking approach to risk management, one that integrates the intertwinedness between energy supply variations, material dependencies, and climate-related hazards into the core of risk assessment and preparedness.

The transformation of global energy systems, driven by fossil fuel decline, critical mineral dependencies, climate pressures, and geopolitical volatility, poses complex, multi-scalar risks to societal resilience. Energy transitions are not merely technological upgrades but deeply systemic shifts that shape the operational landscape of risk management across all sectors. Human systems and critical infrastructure are intricately dependent and therefore vulnerable to energy availability; disruptions, whether abrupt or gradual, can constrain services, generate tensions across economic and political domains, and trigger cascading failures. These vulnerabilities are amplified by climate change, which acts both as a direct hazard and as a feedback mechanism exacerbating systemic fragility. The depletion of fossil fuels^54^ and intensifying competition for the minerals essential to low carbon technologies^70^ further expose global supply chains and emergency systems to new and evolving risks.

Operationalizing this transition requires an expansion of conventional risk management approaches. Tools such as scenario analysis, vulnerability assessments, and adaptive planning frameworks enable practitioners to co-develop and anticipate disruptions and build flexibility into their response systems.^90^^,^^103^ Crucially, these methods must be informed by an understanding of the structural dependencies embedded in energy systems, from geopolitical mineral supply routes to climate-induced volatility in renewable outputs as they are dependent on them.

Looking ahead, cross-sector collaboration will be essential. Risk managers, climate scientists, infrastructure planners, and energy system researchers must co-produce tools, share data, and align priorities to enable resilient infrastructure planning. Areas for future research include improving regional energy-climate models, assessing the resilience of globalized supply chains, and developing governance mechanisms that support the equitable allocation of constrained energy resources. As emphasized by the World Energy Council^104^ and Mitchell et al.^108^ navigating these overlapping risks requires integrated, anticipatory governance. Ultimately, this article offers a call to action: to reframe risk management strategies around the evolving realities of energy system dynamics. The emergence of a new energy paradigm provides an opportunity for risk managers to redefine their strategies in ways that promote sustainability, adaptability, and resilience. By incorporating energy system dynamics, along with their physical, political, and environmental constraints, into resilience frameworks, risk managers can better safeguard essential systems, support effective emergency responses, and foster sustainable pathways through the 21st-century energy landscape. The proactive, multi-faceted approach outlined in this article offers a pathway for navigating the energy transition, ensuring that both infrastructure and communities are prepared for the challenges and opportunities that lie ahead.

## Acknowledgment

Thanks to 10.13039/501100000270NERC (NE/L002485/1) and the London NERC DTP for funding the research program upon which this work is based. Thanks to Dr Ingrid Charvet for her insights, to Danielle Vosper for her insights.

Our sincere and special thanks to the following authors and institutions.

Thanks to the International Energy Agency for allowing us to use their data and work.

Thanks to Jean-Noel Geist and the Shift Project for their work and for allowing us to use their reports for this article.

Thanks to Louis Delannoy for granting us permission to use Figure 4 based on his work.

Thanks to Robert Schaeffer for granting us permission to use his work; Table 1 is derived from it.

Thanks to Stephen Northey for granting us permission to use his data for Figure 7. The figure has been reproduced and adapted from Northey et al., 2014. Data courtesy of Dr Stephen Northey.

## Author contributions

Jonathan Mille has mainly had a role in funding acquisition, conceptualization, investigation, provision of resources, and writing of the original draft preparation and visualization. Danielle Charlton had a role in supervision, provided writing, reviewing and editing, and providing resources. Marleen de Ruiter had a role in writing, reviewing, editing, and providing resources and helped in conceptualization. Raphael Stevens had a role in reviewing, editing and providing extensive resources. Muki Haklay had a role in editing, supervision and supporting conceptualization and validation. Stephen Edwards had a role in supervision, reviewing, editing and validation.

## Declaration of interests

The contact author has declared that none of the authors has any competing interests.

## Declaration of generative AI and AI-assisted technologies in the writing process

During the preparation of this work the author(s) used OPEN AI/Chat GPT4 in order to correct spelling mistakes and reduce repetition. After using this tool/service, the author(s) reviewed and edited the content as needed and take(s) full responsibility for the content of the publication.
